# Technologically Driven Approaches for the Integrative Use of Wild Blackthorn (*Prunus spinosa* L.) Fruits in Foods and Nutraceuticals

**DOI:** 10.3390/antiox12081637

**Published:** 2023-08-19

**Authors:** Oana Viorela Nistor, Ștefania Adelina Milea, Bogdan Păcularu-Burada, Doina Georgeta Andronoiu, Gabriela Râpeanu, Nicoleta Stănciuc

**Affiliations:** Faculty of Food Science and Engineering, Dunărea de Jos University of Galati, Domnească Street 111, 800201 Galati, Romania; Oana.Nistor@ugal.ro (O.V.N.); Adelina.Milea@ugal.ro (Ș.A.M.); Bogdan.Pacularu@ugal.ro (B.P.-B.); Georgeta.Andronoiu@ugal.ro (D.G.A.); Gabriela.Rapeanu@ugal.ro (G.R.)

**Keywords:** wild blackthorn, anthocyanins, antioxidant activity, metabolic syndrome-associated enzyme, jellified blackthorn-based products, nutraceutical

## Abstract

Different technological approaches were used in this study for the valorization of blackthorn (*Prunus spinosa* L.) fruits in marmalade, jam, jelly, and nutraceuticals. Marmalade showed the highest concentrations of polyphenols (7.61 ± 0.05 mg gallic acid equivalents/g dry weight (DW)) and flavonoids (4.93 ± 0.22 mg catechin equivalents/g DW), whereas jam retained the highest content of anthocyanins (66.87 ± 1.18 mg cyanidin-3-*O*-glucoside equivalents/g DW). A good correlation between polyphenol and flavonoid contents and antioxidant activity was found, the highest value being 21.29 ± 1.36 mmol Trolox/g DW for marmalade. Alternatively, the fresh pulp was enriched with inulin, followed by inoculation with *Lactobacillus acidophilus*, and freeze-dried, allowing a powder to be obtained with a viable cell content of 6.27 × 10^7^ CFU/g DW. A chromatographic analysis of blackthorn skin revealed that myricetin (2.04 ± 0.04 mg/g DW) was the main flavonoid, followed by (+)–catechin (1.80 ± 0.08 mg/g DW), (−)-epicatechin (0.96 ± 0.02 mg/g DW), and vanillic acid (0.94 ± 0.09 mg/g DW). The representative anthocyanins were cyanidin 3-*O*-glucoside, cyanidin 3-*O*-rutinoside, and peonidin 3-*O*-glucoside, with an average concentration of 0.75 mg/g DW. The skin extract showed comparable IC50 values for tyrosinase (1.72 ± 0.12 mg/mL), α-amylase (1.17 ± 0.13 mg/mL), and α-glucosidase (1.25 ± 0.26 mg/mL). The possible use of kernels as calorific agents was demonstrated through the evaluation of calorific power of 4.9 kWh/kg.

## 1. Introduction

Nowadays, consumers are more aware of the consequences of a healthy diet, showing a growing interest in the nutritional quality of food. In this context, food manufacturers are challenged to develop new, innovative, and widely attractive foods within a competitive market for healthy foods. Pinacho et al. [1] described two major actions available for the development of a competitive market for functional food. The first one refers to the use of natural, traditional, less-exploited products, such as blackthorn, and the second one involves the production of functional foods and ingredients from unconventional and underutilized raw materials.

Blackthorn (*Prunus spinosa* L.), which belongs to the rose family (Rosaceae), is a perennial plant growing as a shrub on slopes of wild uncultivated areas. The tree is widespread in Romania, growing in plains and in mountainous regions at the edges of forests. The branches are used as infusions for the treatment of hypertension [2]. The main differences between the wild and cultivated varieties are related to the sizes and the seeds of the fruit, and, consequently, the ratio between these two fractions. Therefore, cultivated blackthorn fruits measure 2 cm in diameter, with almost 1 cm of kernel, versus 1 cm diameter and 0.7 cm of kernel for the wild variety. The blackthorn fruits are a rich source of health-related phytochemicals, such as polyphenols, including phenolic acids, flavonols, and anthocyanins [3]. Numerous epidemiological studies have associated the consumption of polyphenol-rich fruits with the prevention of degenerative decline, cardiovascular diseases, and cancer, mostly due to their antioxidant potential [4,5]. Different studies have reported the effect of resveratrol, quercetin, catechin, epicatechin, and caffeic, sinapic, syringic, protocatechuic, and ferulic acids in decreasing the proliferation of certain cell lines in a time- and dose-specific manner [5]. The phytochemical profile of blackthorn highlights its abundance in phenolic compounds, such as quercetin and kaempferol, neochlorogenic, caffeic, and coumarin derivatives, including aesculetin, umbelliferone, scopoletin, anthocyanins, and proanthocyanidins [6]. It is important to mention that according to Pinacho et al. [1], the proanthocyanidins are not often found in nature, their distribution being limited to species from *Ericaceae*, *Hippocastanaceae*, *Lauraceae*, and *Rosaceae*. In a more recent study, the traditional Serbian Prunus fruits, including blackthorn, were evaluated by Popovič et al. [7], reporting their antioxidant capacity, antiproliferative effects in HT29, and inhibition capacity of α-glucosidase, α-amylase, and human dipeptidyl peptidase III (hDPP III) activities.

The blackthorn fruits are blueish black, globular in drupe, and have sizes of 10–15 mm in diameter, with astringent flesh [8]. Although the fruits are far too astringent and harsh tasting for human consumption, despite their succulent appearance, they are not usually eaten as raw fruits but in various processed forms, such as jams, macerated in sugar or honey solutions, and fermented and distilled to obtain a digestive and laxative liquor [9]. These authors suggested that macerated fruits are recommended for gastrointestinal disturbances, diarrhea, dysentery, kidney disease, biliary dyskinesia, gout, whooping cough, and stimulation of digestion [9].

The fruits are quite popular in Romania; however, their high nutritional quality and bioactive compounds are likely not appreciated. Herein, through this study, we intend to develop different integrative technologically driven approaches for the valorization of wild blackthorn (*Prunus spinosa* L.) fruits to be used in foods and nutraceuticals. Therefore, the approach involved the processing of blackthorn by removing the kernels and skins, and then the resulting pulp was used to develop three jellified products, namely, marmalade, jam, and jelly, as well as a nutraceutical product. The jellified blackthorn-based products were analyzed for phytochemical contents in terms of total polyphenolic content; monomeric, co-pigmented, and polymeric anthocyanins; flavonoid content; color change; antioxidant activity; and texture profile analysis. In addition, the pulp was inoculated with *Lactobacillus acidophilus* and freeze-dried in order to develop nutraceuticals with probiotic activities. The resulting powder was evaluated in terms of viable cells. The skins were freeze-dried and characterized for bioactive and inhibitory activity against metabolic syndrome enzymes, such as tyrosinase, α-amylase, and α-glucosidase. The kernels were dried at room temperature in order to be used as a combustion alternative to capitalize upon food waste and to reduce the wastage footprint. This kind of approach is required for food production to optimize labor and disposal costs and to reduce the environmental, economic, and social impacts.

## 2. Materials and Methods

### 2.1. Materials

Fully mature wild blackthorn fruits were purchased from a local gatherer in Buzău, Romania, in September 2021. The fruits were kept in cooled bags for transport to the laboratory, washed with distillated water, and processed immediately. The commercial culture *Lactobacillus acidophilus* was provided by Chr. Hansen (Hoersholm, Denmark). The reagents used to determine total phenolic compounds (TPCs), total flavonoid content (TFC), and total monomeric anthocyanin content (TAC), acetonitrile, formic acid, 2,2-diphenyl-1-picrylhydrazyl (DPPH), 6-hydroxy-2,5,7,8-tetramethylchroman 2-carboxylic acid (Trolox), ethanol, tyrosinase from mushroom (lyophilized powder, ≥1000 unit/mg solid), 3,4-dihydroxy-L-phenylalanine (DOPA), α-glucosidase from *Saccharomyces cerevisiae* (type I, lyophilized powder, ≥10 units/mg protein), p-nitrophenyl-α-d-glucopyranoside, α-amylase from porcine pancreas (type I-A, 700–1400 units/mg protein), sodium phosphate buffer solution (PBS), starch solution, dinitrosalicylic acid (DNS), kojic acid, and acarbose were purchased from Sigma Aldrich (Steinheim am Albuch, Germany). All reagents and solvents were of analytical and HPLC grade.

### 2.2. Fruit Processing

A total of 1000 g of fruits was processed by washing and then blanching in water at 100 °C for 15 min, after which the fruits were crushed. The skins and kernels were removed, and 75% of the pulp was refined for further processing into two blackthorn-based products, namely, marmalade and jam, while juice obtained by squeezing was used for jelly manufacturing. The marmalade was processed by using brown sugar (SanoVita, Valcea, Romania) in a ratio of 2:1 to replace the refined sugar in order to benefit from the health properties of the blackthorn and to obtain functional products. Two hydrocolloids (agar-agar, Biovegan, GmbH Germany, and apple pectin, Medica, Romania) were used to obtain the other two products, namely, jam and jelly. Agar-agar (0.4%) and natural citric acid (0.2%) were used for jam making, while pectin (0.3%) was used for jelly manufacturing. Marmalade, jam, and jelly were obtained via boiling in a multicooker (Philips HD3037/70, 980W, 5 L, Eindhoven, the Netherlands), with continuous stirring, at 100 °C for 1.25 h, 95 °C for 25 min, and 100 °C for 20 min, respectively, to a reasonably thick consistency. The samples were packed in jars, equilibrated at room temperature (21 °C), and stored for 1 week at refrigeration temperature (4 °C) before analysis.

Subsequently, the peels were washed with ultrapure water, dried using paper towel, and freeze-dried (CHRIST Alpha 1-4 LD plus equipment (Germany)) at −42 °C under a pressure of 0.10 mBar for 48 h up to 98% dry weight. Further, the freeze-dried peels were ground using MC 12 equipment (Stephan, Germany) and stored in glass containers with lids in darkness at room temperature until analysis.

### 2.3. Phytochemical Extraction

For the phytochemical characterization of blackthorn products, about 1.0 g of each sample (fresh pulp and processed products) was subjected to solid–liquid solvent-assisted extraction (10 mL of 70% ethanol solution acidified with citric acid (9:1, *v*/*v*)). Further, ultrasound-assisted extraction was performed using a sonication water bath at 40 °C for 30 min, followed by centrifugation at 5000× *g* for 10 min at 4 °C. The supernatants were collected, and the extraction was repeated three times in order to enhance the extraction yield. The collected supernatants were pooled together and used for phytochemical and antioxidant activity analysis. The same procedure was used to extract phytochemicals from freeze-dried skins. Therefore, the extraction was performed by using 1 g of lyophilized skins and 10 mL of 70% ethanol solution acidified with citric acid (9:1, *v*/*v*) four successive times, using sonication with an ultrasonic bath for 30 min at 40 °C, followed by centrifugation at 5000× *g* for 10 min at 4 °C. The four supernatants were collected and concentrated to dryness in a rotary vacuum evaporator. The final extracts were used for chromatographic analysis, phytochemical profiling, and metabolic enzyme inhibitory activity assays.

### 2.4. Total Monomeric Anthocyanin Content (TAC)

The pH-differential method for TAC analysis was applied, following the procedure described by Giusti and Worsltad [10]. Aliquots of 0.2 mL of the supernatants obtained in the extraction step were mixed with 0.8 mL of buffers (pH 1.0 and 4.5, respectively), followed by absorbance reading (Biochrom Libra S22 UV/Vis) at 520 and 700 nm, respectively. TAC content was expressed as milligrams of cyanidin-3-*O*-glucoside equivalents per g dry weight (mg C3G/g DW) according to Equation (1).

(1) $$TAC\left(\frac{mg\;C3G}{g\;DW}\right)=\frac{\left[\left(A_{500_{pH1}}-A_{700_{pH1}}\right)-\left(A_{500_{pH4.5}}-A_{700_{pH4.5}}\right)\right]}{\varepsilon \cdot L}\cdot M_{w}\cdot \frac{M}{V}\cdot D$$

where ε is the molar extinction coefficient for C3G of 26,900 L⋅mol^−1^⋅cm^−1^, L is the cell path length (1 cm), M_W_ is the molecular weight of C3G (449.2 Da), D is the dilution factor, V is the final volume (mL), and M is the samples mass in dry weight (g).

### 2.5. Co-Pigmented, Monomeric, and Polymeric Anthocyanins in Processed Samples

The presence of co-pigmented, monomeric, and polymeric anthocyanins in processed samples was evaluated according to the method described by Tsai and Huang [11]. The method involved the use of 20 µL of 10% (*v*/*v*) acetaldehyde to 2 mL of supernatant, obtained as described above, followed by incubation for 45 min at room temperature and absorbance reading at 520 nm (A_acet_). Further, 260 µL of 5% (*w*/*v*) SO_2_ was added to another 2 mL of supernatant, and the absorbance was read at 520 nm (A_SO2_). The absorbance of the extract (A_extract_) was obtained at 520 nm and multiplied by 10. The percent distribution of the various forms of anthocyanins was calculated using the following equation:

(2) $$\%co\text{-}pigmented=\left[\frac{\left(A_{acet}-A_{extract}\right)}{A_{acet}}\right]\cdot 100$$

(3) $$\%monomeric=\left[\frac{\left(A_{extract}-A_{so2}\right)}{A_{acet}}\right]\cdot 100$$

(4) $$\%polymeric=\left[\frac{\left(A_{SO2}\right)}{A_{acet}}\right]\cdot 100$$

### 2.6. Radical Scavenging Analysis

Aliquots of 0.1 mL of each supernatant were added to 2.9 mL of DPPH solution (0.1 mmol methanolic solution). Discolorations were measured at 515 nm after incubation for 30 min at 25 °C in the dark. Measurements were performed at least in triplicate and expressed as mmol Trolox/g DW using a calibration curve.

### 2.7. HPLC Analysis of Polyphenol Profile in Blackthorn Skin Extracts

An Agilent 1200 HPLC system equipped with an autosampler, degasser, quaternary pump system, multi-wavelength detector (MWD), column thermostat (Agilent Technologies, Santa Clara, CA, USA), and a Synergi Max-RP-80 Å column (250 × 4.6 mm, 4 µm particle size, Phenomenex, Torrance, CA, USA) was used for the separation and identification of the bioactive compounds from the blackthorn extract. The method described by Antoniolli et al. [12] was used for separation of the flavonoids and polyphenolic compounds, with minor modifications. Therefore, two solvent mixtures were used, including solvent A made using a combination of 87:3:10 of ultrapure water, acetonitrile, and formic acid, respectively, and solvent B made of ultrapure water, acetonitrile, and formic acid in a ratio of 40:50:10. For the separation of bioactive compounds, the solvents were flushed into the system with a flow rate of 0.5 mL/min at 30 °C using an injection volume of 20 µL and the following gradient: 0 min—94% A; 20 min—80% A; 35 min—60% A; 40 min—40% A; 45 min—10% A. The method runtime was 80 min, whereas the bioactives were detected at 280 nm and 320 nm. In the case of anthocyanins, the flow rate was 1.0 mL/min for a runtime of 50 min, with detection of wavelengths of 520 nm. To identify the bioactive compounds in the extract, a comparison of the retention times for the obtained peaks was made with the corresponding ones obtained for standard solutions, whereas for quantification, external calibration curves using the peak area were applied. Data acquisition was made using Chemstation software, version B.04.03 (Agilent Technologies, Santa Clara, CA, USA). Results were expressed in mg/100 g DW extract.

### 2.8. Texture Profile Analysis of the Jellified Samples

For the texture profile analysis, the samples were subjected to a double penetration test using a 38.1 mm diameter acrylic cylinder provided by a Brookfield CT3 Texture Analyzer (Brookfield Ametek, Middleboro, MA, USA). The test parameters were set as follows: penetration speed 1 mm/s, target distance 15 mm, trigger load 0.067 N, and load cell 9.8 N. TexturePro CT V1.5 software was used to process the deformation–stress response and to determine textural parameters such as firmness (the maximum force registered for the first penetration cycle, N), adhesiveness (the work required to overcome the attractive forces between the product and the testing probe, mJ), cohesiveness (the measure of how well the product withstands a second deformation relative to its resistance under the first deformation), and springiness (the amount of deformation that is recovered after the first penetration cycle, mm), as defined by Bourne [13]. Three replicates for each sample were conducted.

### 2.9. Color Parameters of the Jellified Samples

The color of the marmalade, jam, and jelly samples was measured using a colorimeter (NR110 3nh, Shenzhen 3nh Technology, Shenzhen, China). The determined parameters were L* (lightness/darkness), a* (red/green), b* (yellow/blue), hue angle (h*), visual color appearance, and chroma (C*) color intensity. Three replicates were carried out for each sample.

### 2.10. Inoculation of Blackthorn Pulp with Lactobacillus acidophilus

About 90 g of fresh pulp was homogenized with 310 mL of distillated water and 1.5% of inulin and allowed to mix on a magnetic stirrer to hydration at 650 rpm overnight. The pH of the mixture was adjusted to 5.6 with 1.0 N NaOH. After homogenization, the mixture was sterilized using a UV lamp and inoculated with 10.5 Log CFU/mL of *L. acidophilus* and freeze-dried (CHRIST Alpha 1-4 LD plus, Osterode am Harz Germany) at −42 °C under a pressure of 10 Pa for 48 h. Afterwards, the powder was collected and packed in metallized bags and kept at 4 °C until further analysis.

### 2.11. The Inhibitory Activity of the Extract on Metabolic-Syndrome-Associated Enzymes

The extract from wild blackthorn skins was tested for potential inhibitory activity on selected metabolic-syndrome-associated enzymes, such as α-glucosidase, α-amylase, and tyrosinase. For the inhibitory experiments, the extract was dissolved in PBS (0.1 M, pH 6.9) at a concentration of 10 mg/mL, and 10-fold dilutions were performed in PBS. For α-glucosidase and α-amylase inhibitory effects, the method described by Meziant et al. [14] was used, whereas for tyrosinase inhibitory activity, the procedure described by Yu et al. [15] was used.

### 2.12. Combustion Characteristics of Kernels

AOAC [16] standardized methods were used to determine the moisture and the relative density of the wild blackthorn kernels. Thermal equivalence was calculated using the conversion coefficients for liquefied petroleum gas (LPG), methane gas, and diesel fuel [17]. The conversion coefficients are presented in Table 1. The higher and the effective calorific power values were calculated using a trial version of a combustion calculator.

### 2.13. Statistical Analysis

The results are expressed in terms of the average of a minimum of three determinations followed by standard deviations. The software Minitab 19 was used to perform the statistical evaluation. The normality (Ryan Joiner test) and homoscedasticity condition of the data were checked. The differences between samples were analyzed using the ANOVA method, and the post hoc analysis was performed based on the Tukey or Games Howell method (when the homoscedasticity condition was not confirmed).

## 3. Results

### 3.1. Phytochemical Characterizations of Blackthorn Pulp

The contents of polyphenolics, flavonoids, and anthocyanins in ethanolic extracts from blackthorn pulp were determined using spectrophotometric methods, which allowed for the measurement of total polyphenolic content (TPC) of 23.34 ± 1.25 mg GAE/g DW and total flavonoid content (TFC) of 20.30 ± 0.17 mg CE/g DW, whereas a higher concentration of TAC of 119.74 ± 6.04 mg C3G/g DW was found (Table 2).

Pinacho et al. [1] studied the total phenolic, flavonoid, and anthocyanin contents of dichloromethane, ethyl acetate, and ethanol, and aqueous extracts of branches, leaves, and fruits from *Prunus spinosa* using spectrophotometric methods. These authors reported higher values of TPC and TFC in fruits (ranging from 184.77 ± 2.11 mg/g when extracted using dichloromethane to 359.11 ± 2.54 mg/g when extracted with ethanol and from 35.51 ± 1.18 mg/g to 141.80 ± 2.11 mg/g, respectively), and comparable TAC up to 179.00 ± 3.61 mg/g. In order to evaluate the antioxidant activity of ethanolic extracts of blackthorn pulp, the method based on the reduction of 2,2-diphenyl-1-picrylhydrazyl (DPPH) allowed an antioxidant activity of 214.48 ± 19.45 mmol Trolox/g DW (Table 2) to be obtained.

The phytochemical contents of the three jellified blackthorn products is given in Table 2. It can be seen that the phytochemical contents highly depended on the processing parameters and added ingredients. The highest TAC was measured in jam, namely, 66.87 ± 1.18 mg C3G/g DW, followed by jelly, with 49.57 ± 1.53 mg C3G/g DW, and marmalade, with 30.62 ± 1.72 mg C3G/g DW. However, the TFC was higher in marmalade (4.93 ± 0.22 mg CE/g DW), followed by jam and jelly (Table 2). The TPC followed the same trend, whereas the antioxidant activity was significant higher in marmalade (21.29 ± 1.36 mmol Trolox/g DW), with no significant differences between jam and jelly. Therefore, it may be appreciated that a positive correlation between TPC and TFC content and antioxidant activity was evidenced for the processed blackthorn products.

In order to understand the heat-induced polymerization of bioactive compounds in processed blackthorn products, the quantification of co-pigmented, polymerized, and monomeric changes in anthocyanin distribution after heating was investigated. As shown in Table 2, monomeric anthocyanin decreased from 99% in the pulp to 92.15 ± 2.33% in marmalade, to 89.74 ± 1.25% in jam, and to 63.01 ± 1.31% in jelly, but there was no co-pigmentation formation in the products during the heating process. Apparently, most of the anthocyanins extracted from blackthorn products existed as monomers, and no intermolecular co-pigmentation between an anthocyanin and a flavonoid or other phenolic compounds through hydrophobic forces and hydrogen bonds took place during heating. The percent of polymeric anthocyanins increased from 7.84 ± 2.57% in marmalade, to 10.25 ± 1.68% in jam, and to 36.98 ± 1.10% in jelly. The proposed mechanism for anthocyanin polymerization during heating has been explained by the hydrolysis of the sugar moiety and decomposition into a chalcone structure, which is accompanied by further transformation into a coumarin glucoside derivative with a loss of B-ring [18]. These authors also reported an increase in polymeric content of anthocyanins during heating of natural and co-pigmented licorice extract with ferulic acid, namely, from 41% to 47%.

### 3.2. Textural Profile Analysis

Texture is very important for consumer acceptance, especially with jellified products, which develop specific textures. The results of texture profile analysis are presented in Table 3.

The highest value of firmness, 0.98 ± 0.06 N, was registered for marmalade, and this could be explained by the denser structure induced by the high content of sugar and fibers [4] and by the intense water evaporation during the manufacturing process. This structure also determined higher attractive forces between the sample and the testing probe, comparing to the other samples, as the adhesiveness value (4.77 ± 0.19 mJ) shows. At the same time, the absence of the jellifying agent in marmalade affected the cohesiveness, this parameter being the lowest (0.44 ± 0.01) for this product. Moreover, marmalade springiness registered a lower value (12.84 ± 0.20 mm) than that of jam (13.34 ± 0.11), proving a more accentuated structure disruption. The jelly, which was obtained from blackthorn juice and pectin, showed the weakest firmness, namely, 0.36 ± 0.02 N, likely due to the lowest content in dry matter. The resulting texture parameters are comparable with those reported by Curi et al. [19] for quince marmalade and Garrido et al. [20] for apple jelly.

### 3.3. Color Parameters

Certain properties are associated with the acceptance of a food, including color, flavor, and emerging health benefits. Much of the interest regards the compounds or treatments that influence the color of the samples. With respect to these, the marmalade, jam, and jelly samples were evaluated by means of color changes. The results of measurements of color parameters L*, a*, and b*, as well as chroma and hue values of the wild blackthorn marmalade, jam, and jelly are presented in Table 4.

The highest value for L* of 20.47 ± 0.05 was measured for marmalade samples, whereas the lowest value of 13.69 ± 0.05 was found for jam, in good agreement with the results reported by Vukoja et al. [21] for cherry jam and Banas et al. [22] for gooseberry and elderberry fruit jams. The darkest sample was the marmalade, which could be attributed to the brown sugar addition and to the prolonged thermal treatment compared to the other two samples. The jelly samples were brighter than the marmalade or jam, due to the translucency of the samples. The highest values for red color (a*), as an indicator of anthocyanin content, were registered for jelly and jam (6.70 ± 0.01 and 6.07 ± 0.15) followed by marmalade (4.41 ± 0.15). The intensity of yellow coloration expressed by the b* parameter ranged between 5.62 ± 0.15 and 7.79 ± 0.04. The values of hue for all the samples were near to the value of 45°, which represents the border between red and pink. As reported by Guiné et al. [23], the chroma values estimated for the processed samples indicate intense color saturation (Table 4).

As expected, the inoculated showed more lightness (L* of 27.77 ± 0.01), due to addition of inulin. The a* (12.88 ± 0.03) and b* (10.23 ± 0.05) parameters suggested a more pronounced tint to red and yellow, respectively (Table 4), when compared to the processed samples. However, the freeze-dried skins showed a negative value for the a* parameter (−6.99 ± 0.01) and a higher value for the b* parameter (33.62 ± 0.01), suggesting an intense red–purple color, with a tint of yellow.

### 3.4. Powder with Probiotic Potential

In order to drive the technological approach for the use of blackthorn fruits into nutraceuticals, the pulp was enhanced with inulin and inoculated with *Lactobacillus acidophilus*. The sample before freeze-drying had an initial inoculum of 1.7 × 10^10^ CFU/g DW, whereas after lyophilization, the powder showed a viable cell content of 6.27 × 10^7^ CFU/g DW. As has been reported, in order to acquire beneficial effects, foods containing probiotics must have at least 10^6^ to 10^7^ live cells per milliliter or gram of the product [24]. The obtained results showed a good retention of the viability of bacteria during freeze-drying, with a satisfactory survival rate, considering that only inulin was used as a protective. In order to increase the retention of *Lactobacillus acidophilus* during freeze-drying, it is important to optimize the formula based on blackthorn phytochemicals as metabolic stimulators and to use different combinations of proteins and polysaccharides to protect lactic acid bacteria [25], whereas the resulting powders can be considered multifunctional, as sources of bioactive, probiotics, and dietary fiber, as well as for color.

### 3.5. Blackthorn Skin as a Source of Bioactive Compounds for Multiple Purposes

**Figure 1:**
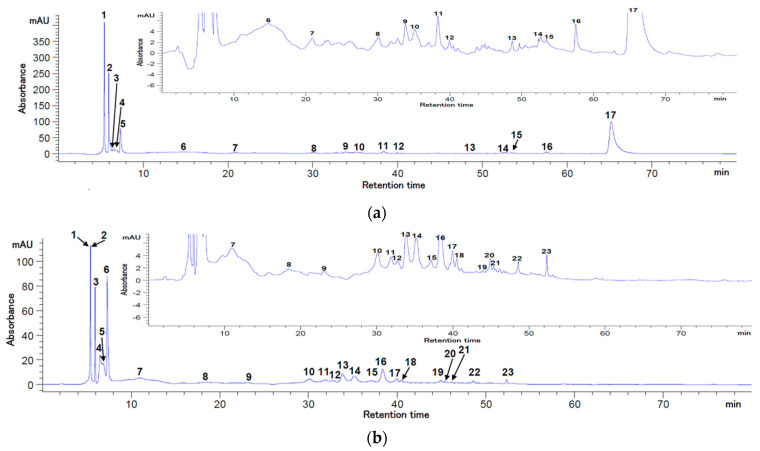
HPLC chromatograms of the flavonoids and polyphenols at 280 nm (**a**) and 320 nm (**b**) from blackthorn extract. Peaks’ identification: (**a**) 1—gallic acid; 2—chlorogenic acid; 3-(-)-epicatechin; 4—caffeic acid; 6—vanillic acid; 7—p-coumaric acid; 12—myricetin; 14—isorhamnetin; 5, 8–11, 13, 15–17—unidentified peaks; (**b**) 1—gallic acid; 2-(+)-catechin; 3—chlorogenic acid; 4—epicatechin; 5—caffeic acid; 17—myricetin; 23—isorhamnetin; 6–16, 18–22—unidentified peaks.

**Figure 2:**
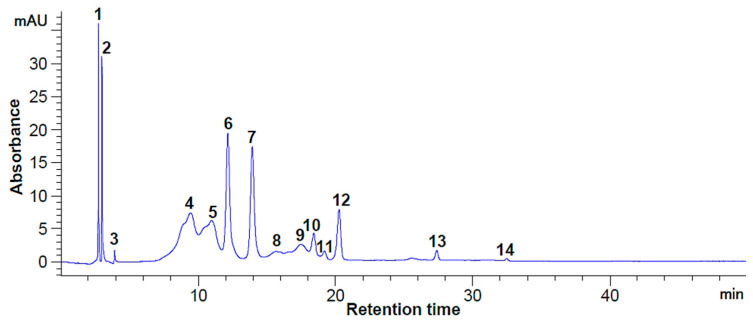
HPLC chromatogram of the anthocyanins from blackthorn extract at 520 nm. Peaks’ identification: 5—Delphinidin 3-*O*-β-D-glucoside; 7—Cyanidin 3-*O*-glucoside; 8—Cyanidin 3-*O*-rutinoside; 10—Peonidin 3-*O*-glucoside; 1–4, 6, 9, 11–14—unidentified peaks.

Given the significant efforts to customize the technological options for superior valorization of by-product streams resulting from blackthorn processing, from a sustainable approach point of view, the skins were freeze-dried and used for phytochemical extractions with the aim of advanced characterization. The HPLC analysis revealed that blackthorn skin can be an important source of bioactive compounds (hydroxycinnamic and hydroxybenzoic acids, flavonoids, and anthocyanins), as depicted in Figure 1 and Figure 2.

Of all the identified bioactive compounds (Table 5), it was observed that the most abundant compound was vanillic acid (943.98 mg/100 g DW extract), followed by chlorogenic acid (689.86 mg/100 g DW extract). Additionally, gallic acid, (−)—epicatechin, and p-coumaric acid concentrations over 300 mg/100 g DW extract were identified at 280 nm. On the contrary, the maximum amount of myricetin and (+)—catechin was determined at 320 nm. The anthocyanins’ profiles presented in Figure 2 showed that the chromatographic conditions used allowed for the separation of five major compounds (peaks 1, 2, 6, 7, 12). Significant concentrations of peonidin and cyanidin 3-*O*-glucosides (ranging from 770.89 to 794.60 mg/100 g DW extract) and cyanidin 3-*O*-rutinoside (712.60 mg/100 g DW) were also found (Table 5).

Our results are in agreement with those reported by Sabatini et al. [26], who identified flavonols and different phenolic acids, such as hydroxycinnamic and hydroxybenzoic acids, including gallic acid, chlorogenic acid, p-coumaric acid, caffeic acid, and vanillic acid derivatives, and also catechin. For example, it has been reported that some bioactive compounds (p-coumaric acid, caffeic acid, catechin, epicatechin) from blackthorn (*Prunus spinosa* L.) fruits or flowers were only identified but not quantified [26,27]. The main anthocyanins identified in this study were cyanidin 3-*O*-glucoside (58.35 mg/100 g), cyanidin 3-*O*-rutinoside (74.25 mg/100 g), and peonidin 3-*O*-glucoside (8.34 mg/100 g) from freeze-dried blackthorn skins. Our results regarding the anthocyanins’ concentrations in the extract showed that they were higher than those previously reported by Popović et al. [28]; the difference could be explained by different blackthorn genotypes [29].

### 3.6. Metabolic Enzymes Inhibitory Potential of Blackthorn Skins Extract

Metabolic syndrome is a collection of metabolic risk factors associated with an increased risk of developing central obesity and inflammation [30]. The abnormal overexpression of tyrosinase it is known to cause a variety of pathological changes, such as pigmentation, light damage, and Parkinson’s disease, which are related to neurodegenerative diseases [31]. The inhibitory activity of the blackthorn extract on tyrosinase reached a maximum value of approximatively 36% at an extract concentration of 10 mg/mL, whereas the IC_50_ value was estimated at 1.72 ± 0.12 mg/mL. Meziant et al. [14] suggested IC_50_ values ranging from 95.08 and 447.49 μg/mL for various flavonoid-rich fig cultivar (*Ficus carica* L.) peel extracts.

The α-glucosidase inhibitory ability reached a maximum of 30% at the maximum concentration tested (10 mg/mL), with IC_50_ value of 1.25 ± 0.26 mg/mL, whereas a similar value was obtained for α-amylase of 1.17 ± 0.13 mg/mL. From the obtained results, it seems that the extract is more effective against α-glucosidase and α-amylase, followed by tyrosinase. The first two enzymes play crucial roles in the hydrolysis of carbohydrates and the production of glucose and therefore in controlling the digestion and absorption of glucose [32]. Numerous studies have demonstrated that the consumption of various biologically active compounds, adjacent to insulin treatment, have the ability to effectively regulate postprandial glycemia and can be used as alternatives to drugs for the development and control of diabetes [33]. Ji et al. [32] suggested IC_50_ values of an anthocyanin-rich bilberry extract of 0.31 ± 0.02 mg/mL for α-glucosidase and 4.06 ± 0.12 mg/mL for α-amylase inhibition, respectively. However, when considering the inhibitory activity of selected enzymes, a comparison with the IC_50_ values of the drugs used in current medical practice can be conducted. Therefore, in our study, acarbose was used as a positive control for the inhibitory activity of α-glucosidase and α-amylase, allowing IC_50_ values of 2.87 ± 0.84 mg/mL and 3.53 ± 0.61 mg/mL, respectively, to be obtained. The corresponding value for kojic acid was 1.19 ± 0.11 mg/mL. The inhibitory values for the blackthorn skin extract were significantly lower when compared to the positive control, suggesting a potential role in controlling a range of metabolic risk factors associated with diabetes and tyrosinase-induced pathological changes.

### 3.7. Kernels as a Source of Combustion

A total of 126 g of kernels was obtained from 1000 g of blackthorn fruits. The moisture content of the kernels was 9%, while the relative density was calculated to be 350 kg/mc. The advantages of reuse and recycling of agricultural waste both in urban and rural areas include high calorific value and low nitrogen, sulfur, and chlorine content [34]. The related ash quantity obtained from the combustion of fruits kernels is also very low (1–1.6%) [35]. Therefore, by using the obtained quantity of kernels, the followed saving could be achieved: 0.063 mc of methane gas, 0.0504 kg LPG, or 0.063 kg diesel. The higher calorific power was calculated as 4.9 kWh/kg, while the effective calorific power ranged between 4.7 and 4.8 kWh/kg.

## 4. Conclusions

This study is an attempt at the integrated, complex valorization of wild blackthorn fruits into three processed products, including marmalade, jam, and jelly, as well as a nutraceutical with probiotic potential, and significant information is provided for health benefits of phytochemicals from blackthorn skins. Moreover, blackthorn kernels may be valorized as a source of heat. The obtained results highlighted that the thermal processing of fresh blackthorn pulp led to a significant decrease in phytochemicals and antioxidant activity. However, marmalade retained the highest phytochemical parameters in terms of total polyphenols, flavonoids, and anthocyanins. Significantly, the anthocyanins remained in a monomeric state, since no co-pigmented and a low rate of polymeric content were found in marmalade when compared to jam and jelly. This phenomenon was correlated with the processing parameters. The pulp was enriched with inulin and inoculated with *Lactobacillus acidophilus* as an attempt to extend the opportunities for blackthorn valorization into nutraceuticals or food-grade ingredients. The presence of viable cells after freeze-drying allows us to conclude that the powder may acquire beneficial probiotic effects. However, additional studies are needed in order to optimize the formula and to benefit, concomitantly, from the recognized potential of polyphenols to stimulate the metabolic activity of probiotics. Blackthorn skin was found to be a valuable source of polyphenols, with remarkable contents of gallic acid, chlorogenic acid, (−)—epicatechin, caffeic acid, vanillic acid, p-coumaric acid, myricetin, isorhamnetin, (+)—catechin, delphinidin 3-*O*-β-D-glucoside, cyanidin 3-*O*-glucoside, cyanidin 3-*O*-rutinoside, and peonidin 3-*O*-glucoside. The extract showed efficient inhibitory activity against tyrosinase, α-amylase, and α-glucosidase, being a valuable candidate for different applications in foods and pharmaceuticals. The potential for using kernels as heating agents was made possible through the evaluation of calorific power.

## Figures and Tables

**Table 1:** Conversion coefficients of the main classical fuels.

1 kg of LPG (liquefied petroleum gas)	2.5 kg of fruit kernels
1 kg of methane gas	2 kg of fruit kernels
1 kg of diesel	2 kg of fruit kernels

**Table 2:** The phytochemical profile of the wild blackthorn pulp processed products.

Samples	Unprocessed Pulp	Marmalade	Jam	Jelly
Total monomeric anthocyanin content (mg C3G/g DW)	119.74 ± 6.04 ^a^	30.62 ± 1.72 ^c^	66.87 ± 1.18 ^b^	49.57 ± 1.53 ^bc^
Total polyphenolic content (mg GAE/g DW)	23.34 ± 1.25 ^a^	7.61 ± 0.05 ^b^	5.32 ± 0.15 ^c^	5.76 ± 0.97 ^c^
Total flavonoid content (mg CE/g DW)	20.30 ± 0.17 ^a^	4.93 ± 0.22 ^b^	3.11 ± 0.09 ^c^	3.09 ± 0.10 ^c^
Antioxidant activity (mmol Trolox/g DW)	214.48 ± 19.45 ^a^	21.29 ± 1.36 ^b^	10.10 ± 2.54 ^b^	10.78 ± 0.74 ^b^
Monomeric anthocyanins, %	99.00 ± 0.5 ^a^	92.15 ± 2.33 ^b^	89.74 ± 1.25 ^b^	63.01 ± 1.31 ^c^
Polymeric anthocyanins, %	0 ^c^	7.84 ± 2.57 ^b^	10.25 ± 1.68 ^b^	36.98 ± 1.10 ^a^

Values are represented as mean ± standard errors. Means in a row sharing different superscript letters (a, b, c) are statistically significantly different (*p* < 0.05).

**Table 3:** Texture profile analysis of wild blackthorn marmalade, jam, and jelly.

Color Parameters	Marmalade	Jam	Jelly
Firmness, N	0.98 ± 0.06 ^a^	0.57 ± 0.02 ^a^	0.36 ± 0.02 ^a^
Adhesiveness, mJ	4.77 ± 0.19 ^a^	3.49 ± 0.26 ^a^	1.46 ± 0.01 ^a^
Cohesiveness, -	0.44 ± 0.01 ^a^	0.72 ± 0.03 ^a^	0.62 ± 0.03 ^a^
Springiness, mJ	12.84 ± 0.20 ^a^	13.34 ± 0.11 ^a^	10.27 ± 0.76 ^a^

Values are represented as mean ± standard errors. Means in a row bearing the same superscript letters do not differ significantly with a *p*-value of 0.999.

**Table 4:** Color parameters of wild blackthorn marmalade, jam, and jelly.

Color Parameters	Marmalade	Jam	Jelly	Probiotic Powder	Freeze-Dried Skins
L*	20.47 ± 0.05 ^a^	17.13 ± 0.03 ^a^	13.69 ± 0.05 ^a^	27.77 ± 0.01 ^a^	16.65 ± 0.02 ^a^
a*	4.41 ± 0.15 ^a^	6.07 ± 0.15 ^a^	6.79 ± 0.01 ^a^	12.88 ± 0.03^a^	−6.99 ± 0.01 ^a^
b*	7.79 ± 0.04 ^a^	6.00 ± 0.20 ^a^	5.62 ± 0.15 ^a^	10.23 ± 0.05 ^a^	33.62 ± 0.01 ^a^
C*	10.40 ± 0.1 ^a^	8.09 ± 0.01 ^a^	7.16 ± 0.01 ^a^	16.58 ± 0.03 ^a^	34.35 ± 0.05 ^a^
h*	51.43 ± 0.01 ^a^	46.60 ± 0.05 ^a^	49.40 ± 0.03 ^a^	38.91 ± 0.01 ^a^	101.71 ± 0.04 ^a^

L* (lightness/darkness), a* (red/green), b* (yellow/blue), h* (hue angle), C* (chroma, color intensity). Values are represented as mean ± standard errors. Means in a row sharing the same superscript letters do not differ significantly with a *p* value of 0.999.

**Table 5:** Concentrations of bioactive compounds identified by HPLC.

Bioactive Compound	Concentration, mg/100 g DW Extract	
	280 nm	320 nm
Gallic acid	377.00 ± 40.62	123.25 ± 2.02
Chlorogenic acid	689.86 ± 65.44	74.66 ± 2.47
(−)—Epicatechin	393.41 ± 30.19	969.16 ± 19.29
Caffeic acid	22.80 ± 0.65	40.42 ± 2.65
Vanillic acid	943.98 ± 91.27	nd
p-coumaric acid	364.56 ± 7.83	nd
Myricetin	324.45 ± 39.65	2041.20 ± 46.13
Isorhamnetin	16.44 ± 0.06	9.86 ± 0.29
(+)—Catechin	nd	1808.00 ± 85.84
Delphinidin 3-*O*-β-D-glucoside	316.14 ± 0.09	
Cyanidin 3-*O*-glucoside	794.60 ± 0.19	
Cyanidin 3-*O*-rutinoside	712.60 ± 0.18	
Peonidin 3-*O*-glucoside	770.89 ± 0.01	

Reported results are average values of duplicate measurements (*n* = 2) followed by standard deviations; nd—not determined.

## Data Availability

Data are available upon request.

## References

[1] Pinacho R., Cavero R.Y., Astiasarán I., Ansorena D., Calvo M.I. (2015). Phenolic compounds of blackthorn (*Prunus spinosa* L.) and influence of in vitro digestion on their antioxidant capacity. J. Funct. Foods.

[2] Calvo M.I., Cavero R.Y. (2014). Medicinal plants used for cardiovascular diseases in Navarra and their validation from Officinal sources. J. Ethnopharmacol..

[3] Acero N., Gradillas A., Beltran M., García A., Mũnoz Mingarro D. (2019). Comparison of phenolic compounds profile and antioxidant properties of different sweet cherry (*Prunus avium* L.) varieties. Food Chem..

[4] Sikora E., Bieniek M.I., Borczak B. (2013). Composition and antioxidant properties of fresh and frozen stored blackthorn fruits (*Prunus spinosa* L.). Acta Sci. Pol. Technol..

[5] Kampa M., Nifli A.-P., Notas G., Castanas E. (2007). Polyphenols and cancer cell growth. Rev. Physiol. Biochem. Pharmacol..

[6] Ruíz-Rodríguez B.M., de Ancos B., Sánchez-Moreno C., Fernández-Ruíz V., Sánchez-Mata M.C., Cámara M., Tardío J. (2014). Wild blackthorn (*Prunus spinosa* L.) and hawthorn (*Crataegus monogyba* Jacq.) fruits as valuable sources of antioxidants. Fruits.

[7] Popovič B.M., Blagojevič B., Kucharska A.Z., Agič D., Magazin N., Milovič M., Serra A.T. (2021). Exploring fruits from genus Prunus as a source of potential pharmaceutical agents—In vitro and in silico study. Food Chem..

[8] Marakoğlu T., Arslan D., Ozcan M., Hacıseferoğulları H. (2005). Proximate composition and technological properties of fresh blackthorn (*Prunus spinosa* L.) subsp *dasyphylla* (Schur.)) fruits. J. Food Eng..

[9] Barros L., Carvalho A.M., Morais J.S., Ferreira I.C.F.R. (2010). Strawberry-tree, blackthorn and rose fruits: Detailed characterization in nutrients and phytochemicals with antioxidant properties. Food Chem..

[10] Giusti M.M., Worsltad R.E. (2001). Characterization and measurement of anthocyanins by UV visible spectroscopy. Current Protocols in Food Analytical Chemistry.

[11] Tsai P.-J., Huang J.P. (2004). Effect of polymerization on the antioxidant capacity of anthocyanins in Roselle. Food Res. Int..

[12] Antoniolli A., Fontana A.R., Piccoli P., Bottini R. (2015). Characterization of polyphenols and evaluation of antioxidant capacity in grape pomace of the cv. Malbec. Food Chem..

[13] Bourne M.C. (2002). Principles of objective texture measurement. Food Texture and Viscosity.

[14] Meziant L., Bachir-bey M., Bensouici C., Saci F., Boutiche M., Louaileche H. (2021). Assessment of inhibitory properties of flavonoid-rich fig (*Ficus carica* L.) peel extracts against tyrosinase, α-glucosidase, urease and cholinesterases enzymes, and relationship with antioxidant activity. Eur. J. Integr. Med..

[15] Yu Q., Fan L., Duan Z. (2019). Five individual polyphenols as tyrosinase inhibitors: Inhibitory activity, synergistic effect, action mechanism, and molecular docking. Food Chem..

[16] AOAC (1990). Official Methods of Analysis 15th Edition of the Association of Official Analytical Chemists.

[17] (2012). Solid Biofuels—Fuel Specifications and Classes—Part 6: Non-Woody Pellets for Non-Industrial Use.

[18] Chitgar M.H., Aalami M., Kadkhodaee R., Maghsoudlou Y., Milani E. (2018). Effect of thermosonication and thermal treatments on phytochemical stability of barberry juice copigmented with ferulic acid and licorice extract. Innov. Food Sci. Emerg. Technol..

[19] Curi P.N., Coutinho G., Matos M., Pio R., Albergaria F.C., de Souza V.R. (2018). Characterization and Marmelade processing potential of quince cultivars cultivated in tropical regions. Rev. Bras. Frutic..

[20] Garrido J.J., Lozano I.E., Genovese D.B. (2015). Effect of formulation variables on rheology, texture, colour, and acceptability of apple jelly: Modelling and optimization. LWT—Food Sci. Technol..

[21] Vukoja J., Pichler A., Kopjar M. (2019). Stability of anthocyanins, phenolics and color of tart cherry jams. Foods.

[22] Banas A., Korus A., Korus J. (2018). Texture, Color, and Sensory Features of Low-Sugar Gooseberry Jams Enriched with Plant Ingredients with ProHealth Properties. J. Food Qual..

[23] Guiné R.P.F., Correia P.M.R., Florença S.G. (2018). Development of jelly gums with fruits and herbs: Colour and sensory evaluation. Int. J. Environ. Res. Public Health.

[24] Tripathi M.K., Giri S.K. (2014). Probiotic functional foods: Survival of probiotics during processing and storage. J. Funct. Foods.

[25] Nale Z., Tontul I., Aşçi Arslan A., Sahin Nadeem H., Kucuk-cetin A. (2018). Microbial viability, physicochemical and sensory properties of kefir microcapsules prepared using maltodextrin/arabic gum mixes. Int. J. Dairy Technol..

[26] Sabatini L., Fraternale D., Di Giacomo B., Mari M., Albertini M.C., Gordillo B., Rocchi M.B.L., Sisti D., Coppari S., Semprucci F. (2020). Chemical composition, antioxidant, antimicrobial and anti-inflammatory activity of *Prunus spinosa* L. fruit ethanol extract. J. Funct. Foods.

[27] Popović B.M., Blagojević B., Ždero Pavlović R., Mićić N., Bijelić S., Bogdanović B., Mišan A., Duarte C.M.M., Serra A.T. (2020). Comparison between polyphenol profile and bioactive response in blackthorn (*Prunus spinosa* L.) genotypes from north Serbia-from raw data to PCA analysis. Food Chem..

[28] Marchelak A., Olszewska M.A., Owczarek A. (2020). Data on the optimization and validation of HPLC-PDA method for quantification of thirty polyphenols in blackthorn flowers and dry extracts prepared thereof. Data Brief.

[29] Marchelak A., Olszewska M.A., Owczarek A. (2020). Simultaneous quantification of thirty polyphenols in blackthorn flowers and dry extracts prepared thereof: HPLC-PDA method development and validation for quality control. J. Pharm. Biomed. Anal..

[30] Aboonabi A., Meyer R.R., Gaiz A., Singh I. (2020). Anthocyanins in berries exhibited anti-atherogenicity and antiplatelet activities in a metabolic syndrome population. Nutr. Res..

[31] Jia Y.L., Zheng J., Yu F., Cai Y.X., Zhan X.L., Wang H.F., Chen Q.X. (2016). Anti-tyrosinase kinetics and antibacterial process of caffeic acid N-nonyl ester in Chinese Olive (*Canarium album*) postharvest. Int. J. Biol. Macromol..

[32] Ji Y., Liu D., Jin Y., Zhao J., Zhao J., Li H., Li L., Zhang H., Wang H. (2021). In vitro and in vivo inhibitory effect of anthocyanin-rich bilberry extract on α-glucosidase and α-amylase. LWT—Food Sci. Technol..

[33] Sohretoglu D., Sari S., Barut B., Ozel A. (2018). Discovery of potent alpha-glucosidase inhibitor flavonols: Insights into mechanism of action through inhibition kinetics and docking simulations. Bioorganic Chem..

[34] Dołzynska M., Obidzinski S., Kowczyk-Sadowy M., Krasowska M. (2019). Densification and Combustion of Cherry Stones. Energies.

[35] Bryś A., Zielińska J., Głowacki S., Tulej W., Bryś J. (2020). Analysis of possibilities of using biomass from cherry and morello cherry stones for energy purposes. E3S Web Conf..

